# Social Media Use Among People Living with Chronic Pain: Cross-Sectional Study of Associated Factors

**DOI:** 10.2196/88412

**Published:** 2026-09-04

**Authors:** Alexandra Martel, Paul Farand, Éloïse Farand, Valérie St-Pierre, Anaïs Lacasse, CEMPUS Cohort Research Group

**Affiliations:** 1 Département de médecine, Faculté de médecine et des sciences de la santé de l’Université de Sherbrooke et Centre de recherche clinique du Centre intégré universitaire de santé et de services sociaux de l’Estrie, Centre hospitalier universitaire de Sherbrooke, Sherbooke, QC, Canada; 2 Service de cardiologie, Département de médecine, Faculté de médecine et des sciences de la santé de l’Université de Sherbrooke et Centre de recherche clinique du Centre intégré universitaire de santé et de services sociaux de l’Estrie, Centre hospitalier universitaire de Sherbrooke, Sherbooke, QC, Canada; 3 Département d'anesthésiologie, Faculté de médecine et des sciences de la santé de l’Université de Sherbrooke et Centre de recherche clinique du Centre intégré universitaire de santé et de services sociaux de l’Estrie, Centre hospitalier universitaire de Sherbrooke, Sherbooke, QC, Canada; 4 Département des sciences de la santé, Université du Québec en Abitibi-Témiscamingue, 445, boul. de l'Université, Rouyn-Noranda, QC, J9X 2L7, Canada, 1 8197620971 ext 2722; 5 See Acknowledgments

**Keywords:** chronic pain, social media, prevalence of use, associated factors, determinants, recruitment, knowledge mobilization, knowledge translation

## Abstract

**Background:**

Social media is increasingly used for research recruitment and knowledge mobilization. However, the general use of social media has not been documented among individuals living with chronic pain.

**Objective:**

Our study aimed to describe social media use in this population and identify associated factors.

**Methods:**

This cross-sectional study analyzed data from 1549 participants of the CEMPUS (Cohort as Part of Undergraduate Medical Studies at the University of Sherbrooke) cohort (Quebec, Canada) who reported living with chronic pain and completed a health questionnaire either online or by phone in 2024. Multivariable logistic regression was conducted to examine factors associated with social media use.

**Results:**

Overall, 86.4% (n=1308) of participants (95% CI 84.7‐88.1) reported using social media. Use was higher among females (n=853, 88.9%) than males (n=446, 81.7%). The most frequently used platforms were Facebook, Messenger, and YouTube. Factors associated with higher odds of reporting social media use included being a female (adjusted odds ratio [aOR] 1.96, 95% CI 1.39‐2.77), having a postsecondary education (aOR 1.57, 95% CI 1.11‐2.23), drinking alcohol (occasional vs never: aOR 1.64, 95% CI 1.04‐2.57), and reporting difficulties accessing health care (aOR 1.50, 95% CI 1.01‐2.22). Lower odds of reporting social media use were associated with older age (aOR 0.94, 95% CI 0.92‐0.96), having chronic pain for 10 years or more (aOR ≥10 vs <1 y=0.55, 95% CI 0.31‐0.96), and having higher levels of depression (aOR 0.92, 95% CI 0.87‐0.99).

**Conclusions:**

Most participants with chronic pain use social media; this points to the potential of social media for research recruitment and knowledge mobilization. However, as exclusive reliance on these platforms may overlook certain groups, there is a clear need for diverse outreach strategies.

## Introduction

Among the general population, 1 in 5 people lives with chronic pain [1,2], which is defined as pain that persists or recurs for more than 3 months, as per the most recent recommendations [3]. This proportion represents more than 7.6 million Canadians [4]. As proposed in the biopsychosocial model of pain [5], biological, psychological, and social factors specific to each individual modulate this experience. Chronic pain is an invisible condition that was recognized as a disease in itself in the latest version of the World Health Organization’s (WHO) *International Classification of Diseases* [6]. It disproportionately impacts different groups (eg, women), and roughly 1 in 3 Canadians aged 65 years and older lives with chronic pain [4].

Although our understanding of chronic pain is continually progressing, there remain gaps in pain research (eg, factors accounting for the transition of acute to chronic pain, tailoring chronic pain treatments to individual patients, and the process of pain resolution) [4,7,8]. Further research could employ different participant recruitment strategies such as flyers in clinics, newspaper advertisements, radio and television broadcasts, letters, emails, website listings, word-of-mouth recruitment, and snowball sampling [9]. With advancements in technology, social media “as a strategy” is increasingly used [10]. Indeed, previous studies have relied on social media to recruit a large number of participants living with chronic pain in just a few weeks [11-13]. Social media is defined as online and mobile services that enable users to participate in online discussions, create content with other users, or connect with online communities [14]. In a scoping review of medical research studies using social media–based recruitment (not specific to the field of pain), Facebook was the most widely used platform (28 out of 30 studies) [15]. Social media is also used for knowledge mobilization activities, which encourage the application of research knowledge in real-world practice [4]. One such activity, the #*ItDoesntHavetoHurt* social media campaign, disseminates evidence-based information about children’s pain to parents [16]. Social media can also be used to advertise web-based chronic pain self-management programs.

The use of social media for the recruitment of research participants has certain disadvantages, including issues of representativeness, data security concerns, and access restrictions [17]. Conversely, it also offers many advantages: reaching large audiences without geographic borders, making contact with hard-to-reach populations, recruiting individuals from community settings rather than being limited to clinical populations receiving care, targeting specific groups, conducting rapid recruitment, encouraging the active involvement of participants, and lowering recruitment process costs, to name a few [10,17,18]. In a literature review, Darko et al [10] reported that the cost per person of social media recruitment methods ranged from US $1.78 to US $292.08, while the cost per person of other recruitment methods ranged from US $2.08 to US $2761.99. Given the efficiency of social media in reaching broad audiences in very little time [19], such a strategy can overcome the limitations of traditional recruitment and knowledge mobilization methods. That said, when employing social media, it is important to consider whether the target audience matches the actual audience being reached. Could such recruitment approaches inadvertently yet systematically exclude certain people living with chronic pain?

In the literature, social media use among people living with chronic pain ranges from 38.1% to 90% [20-23]; older studies (2013: 38.1%; 2015: 90%) and more recent studies (2022: 68.1%; 2023: 81.6%) have reported similarly variable rates. However, none of these studies examined the general use of social media in this population; rather, they assessed the use of social media for specific purposes, including obtaining health information [23], self-management of pain [21], seeking a diagnosis of endometriosis or learning about the condition [20], or using digital health resources [22]. Most studies focused on specific populations (eg, people with endometriosis [20], individuals aged 21-64 years with a self-identified chronic pain diagnosis involved in the *PatientsLikeMe* platform [22], or individuals with chronic low back pain followed by a rheumatology service [23]). Other studies focused on specific social media platforms (eg, Twitter or Instagram) to analyze pain-related social media posts [24,25]. To our knowledge, no study has examined the sociodemographic and health factors associated with general social media use in people living with chronic pain. Thus, the objectives of this study were to (1) describe the general use of social media among people living with chronic pain (prevalence) and (2) to identify the sociodemographic and health factors associated with social media use among this population. Developing a better understanding of the recruitment methods of people living with chronic pain for research purposes and establishing how to reach such people for knowledge mobilization activities align with the Canadian Pain Task Force Action Plan for Pain in Canada [26], particularly its fourth and fifth goals. These goals aim to ensure knowledge mobilization in practice, strengthen future research capabilities, improve the collection of pain data nationwide, and include a broader range of populations disproportionately impacted by chronic pain in research [26]. We hypothesize that while social media is commonly used by people living with chronic pain, some sociodemographic and health-related groups may be underrepresented in such recruitment and knowledge mobilization efforts.

## Methods

The reporting of this paper adheres to the STROBE (Strengthening the Reporting of Observational Studies in Epidemiology) statement for cross-sectional studies [27] and the CHERRIES (Checklist for Reporting Results of Internet E-Surveys) [28].

### Study Design and Selection Criteria

A cross-sectional study was conducted using data from the CEMPUS (Cohort as Part of Undergraduate Medical Studies at the University of Sherbrooke) cohort. Described elsewhere [29], the CEMPUS cohort was designed to assist undergraduate medical students in their research training and to help them acquire a better understanding of the health status and factors influencing the health of Canadians. Most of the survey content was inspired by questions from Statistics Canada’s Canadian Community Health Survey [30]. Since 2019, CEMPUS has been conducted each year. In the 2024 cycle, participants completed a survey online (1848/2887, 64%) or via a phone interview (1039/2887, 36%), depending on their preference. Data collection occurred between August 25 and September 25, 2024 (N=2887). A convenience sampling method was used.

Eligibility criteria were as follows: participants had to be reachable by phone or electronically, they had to be able to answer a questionnaire in French or English, and they had to reside in the province of Quebec (Canada). The target audience consisted of people who had agreed to be contacted again in the following prior surveys: (1) a survey conducted by the public health department of Estrie, that is, *Enquête de santé populationnelle estrienne* [31], or (2) previous CEMPUS cycles. Another eligibility criterion for this study was self-reported chronic pain (ie, pain for at least 3 mo). As a result, we selected 1549 participants from the CEMPUS cohort.

### Data Collection

Usability and technical functionality were maximized through prior iterations of the CEMPUS survey and pretesting conducted before each survey cycle. Third-year medical students at the Université de Sherbrooke were responsible for developing the questionnaire and collecting the data. Interviewers who had received standardized training contacted potential participants by telephone, read the information required to allow free and informed consent, and determined participants’ preferred mode of questionnaire completion. The questionnaire was then either administered by telephone, with verbal consent electronically documented by the interviewer and responses entered in real time into REDCap, or self-administered on the same platform through a secure form sent to participants by email that included an electronic consent checkbox.

### Ethical Considerations

The survey was voluntary. Participants provided their informed consent online or via telephone interviews while completing the survey, after being informed of the study objectives, the members of the research team, the approximate duration of the questionnaire, and the procedures for data storage and retention, including the secure storage of study data on the University of Sherbrooke’s servers, which are protected by multifactor authentication. No incentives were offered. Participants could proceed without answering every question. The human research ethics committee of the CIUSSS-Estrie-CHUS, the CEMPUS principal investigator’s institution (PF; approval number MP-31-2029-3172; amendment form F1H-68285), approved the study protocol, along with the sharing of the anonymized database with members of the team.

Participants were not required to register or create an account before completing the survey. The CEMPUS targeted recruitment approach (using previous survey participants) reduced the risk of duplicate and fraudulent responses (eg, bots), a challenge increasingly reported in web-based surveys that rely on social media recruitment [32]. It should also be noted that approximately one-third of participants completed the questionnaire via telephone interviews, which further limited these risks. For these reasons, no responses were excluded from the study dataset. In our study, a survey response rate specific to individuals living with chronic pain could not be calculated because chronic pain status was unknown among the individuals contacted through the recontact lists of previous survey participants. Consequently, the number of eligible individuals living with chronic pain was unavailable, precluding the determination of an appropriate denominator. More broadly, an exact response rate for the CEMPUS cohort was difficult to calculate because, although recruitment was based on existing contact lists, contact information may have changed over time and some individuals may no longer have been eligible (eg, because they had passed away), making it impossible to establish an accurate denominator. However, characteristics of our sample can be compared to representative samples of individuals living with chronic pain to evaluate representativeness. The sex assigned at birth and employment characteristics of this sample of individuals living with chronic pain were found to be comparable to other Canadian and international probability samples of such people [33-35]. Postsecondary education rates were also similar to those in probabilistic samples (a difference of <10 percentage points; 74.5% vs 67.6% [35]). However, within the CEMPUS sample of people living with chronic pain, the mean age was higher than in other random samples (61 vs 46.6‐49.9 years old [33-35]). People with a pain duration of 10 years or more were also underrepresented when compared with 2 probabilistic samples (28.4% vs 46%‐46.7% [33,36]). This highlights the importance of first stratifying prevalence results by age and pain duration in chronic pain studies using CEMPUS data and then applying necessary multivariable adjustments to account for underrepresentation and overrepresentation.

### Study Variables

The CEMPUS cohort questionnaire included over 140 items organized into 12 blocks or pages; however, only a subset of items relevant to the objectives of the present study was analyzed.

#### Chronic Pain Variables

The survey section on chronic pain included 7 items. Participants were asked if they had experienced any kind of pain for at least 3 months and if their pain was constant or occasional. A third item also asked participants to report the duration of their pain. The last 4 items of the chronic pain section represented the PROMIS (Patient-Reported Outcomes Measurement Information System) Short Form v1.1–Pain Interference 4a [37]. The PROMIS score ranges from 4 to 20 and is calculated with the 4 items answered on a 5-point Likert scale. The instrument evaluates pain interference in the everyday life of participants in the past 7 days (ie, day-to-day activities, work around the home, ability to participate in social activities, and household chores).

#### Social Media Use

The average time per day spent on social media was reported by participants for 3 purposes (ie, directly connecting with other people, recreational use without directly interacting with people, and professional use). In our study, the percentage of participants who reported time spent on social media for any purpose was calculated to evaluate the overall prevalence of social media use (% with at least some minutes of use). The survey section on social media also included the 6 items from the Bergen Social Media Addiction Scale (BSMAS) [38-40]. The BSMAS score (range 6‐30) was calculated by adding the 6 items answered on a 5-point Likert scale to measure problematic social media use among participants in the past year. Dichotomous variables for “problematic social media use” were also created, based on different BSMAS cutoff points reported in the literature (score ≥19/30 [41] or ≥24/30 [38,42]). Participants were also asked in an open-ended question to name the social media platforms they used at least once a week in the past year. We compiled the 12 most reported platforms to measure their prevalence of use in our study sample.

#### Covariables

The following covariables were considered: (1) sociodemographic characteristics: age, sex assigned at birth (female vs male), gender identity (woman, man, and gender-diverse), country of birth (Canada vs other), employment (yes vs no), postsecondary education (yes vs no), and household income (dichotomized as higher vs lower or close to the median household income in the province of Quebec, which was approximately $62,900 (USD $45,600) in 2022 [43]); (2) chronic pain characteristics: pain frequency (continuous vs intermittent), pain duration (categorized as 3-11 mo, 1-4 y, 5-9 y, and ≥10 y), and pain interference based on the PROMIS score [37]; (3) health profile and lifestyle variables: average time per day spent sitting down, sleep difficulties according to the Insomnia Severity Index [44-46], anxiety and depression levels according to the Hospital Anxiety and Depression Scale (HADS) [47-49], number of comorbidities, current tobacco smoking (yes vs no), alcohol consumption of five drinks or more on a single occasion (never vs occasionally, defined as 3 times per month and less, vs regularly, defined as once a week or more), cannabis smoking in the last year (yes vs no), and difficulties accessing health care or advice in the last year (yes vs no). All participants received the survey items in the same order.

### Statistical Analysis

Descriptive statistics were used to summarize the characteristics of the study population and the social media use variables; numbers and proportions were used for categorical variables, while means, SDs, medians, and IQRs were used for continuous variables. We compared the prevalence of the general use of social media across the following categories: sex at birth, age, and pain duration. To align with sex and gender analysis guidelines [50], the prevalence should also be compared across gender identity subgroups. However, in this study, given that the gender diversity subgroup was small (n=3), we used only sex at birth as a stratification variable and potential determinant. We selected age and pain duration as stratification variables because, as noted above, the CEMPUS cohort likely overrepresents older individuals and underrepresents those with a pain duration of 10 years or more.

To achieve the second objective of this study, we used a multivariable logistic regression model to examine the association between sociodemographic and health factors (independent variables) and social media use (dichotomous dependent variable). We favored logistic regression over linear regression (eg, using the number of hours on social media as a dependent variable) for ease of interpretation. All covariates were preselected a priori based on the scientific literature [51-54] and clinical judgment. Following recommendations [55], we opted for this approach over criticized selection methods, such as relying on bivariate regression *P* values or stepwise procedures [55,56]. A parsimonious modeling strategy was adopted, given the relatively small number of individuals in the smallest outcome category (n=209). Categories were combined when appropriate based on similarity in their association with the outcome (eg, postsecondary vs no postsecondary education). Consistent with common practices in logistic regression, the number of events in the smallest outcome category was used to inform the maximum number of dummy variables included (≈10 events per variable) [57], corresponding to a model with no more than 20 to 21 dummy variables. Our logistic regression model was developed as an explanatory model aimed at identifying factors independently associated with social media use rather than predicting which individuals would or would not use social media. In this context, the primary interest lies in the magnitude and direction of the associations rather than in the model’s ability to correctly classify individuals. Our study reported adjusted odds ratios (aORs) that assess the association between the independent variables and the odds of using social media, their *P* values, and their 95% CIs. We assessed model quality by confirming that CIs were not excessively wide, assessing multicollinearity (variance inflation factors <5), and testing overall fit using the Hosmer-Lemeshow test (*P*>.05) [56,58]. Missing data were low for all variables included in the multivariable model (ranging from 0% to 2.3%), except for household income (10.3%), number of comorbidities (11.4%), and pain duration (14.1%). We thus replaced missing observations using a multiple imputation approach based on fully conditional specification [59], generating 5 imputed datasets. The resulting estimates were combined according to the Rubin rules to obtain the final pooled results. We performed 10 iterations of the fully conditional specification algorithm to ensure adequate convergence of imputations for continuous and categorical variables. All analyses were performed using IBM SPSS Statistics version 31.

It should be noted that a BSMAS score of 19 or more (ie, a cutoff score indicating “problematic social media use” in the literature [41]) and an average time spent on social media for recreational purposes of 420 minutes per day or more (ie, a cutoff value our research team considered problematic, as it represents a 7-h workday) were 2 other dependent variables initially considered for the multivariable analysis. However, these variables were not used as dependent variables of interest because too few participants belonged to these subgroups (n=27 and n=12).

## Results

**Figure 1.**
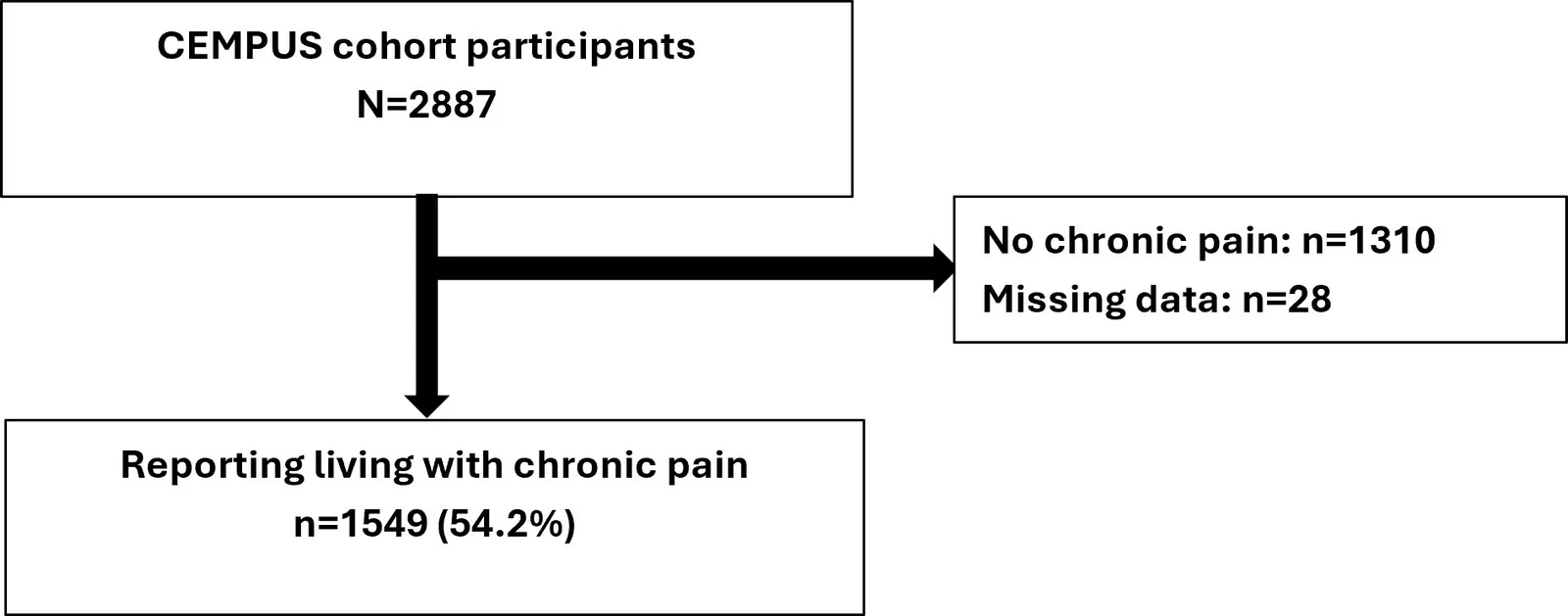
Study sample selection. The valid percentage was calculated after listwise deletion of cases with missing data. CEMPUS: Cohort as Part of Undergraduate Medical Studies at the University of Sherbrooke.

Among the 2887 participants of the CEMPUS cohort, 2859 had complete information on pain variables, and 1549 (54.2%) participants were identified as living with chronic pain (Figure 1). Table 1 shows the characteristics of the sample. The average age was 61.1 (SD 14.4) years, and most participants were 55 years of age or older (n=1090, 70.4%). Among the sample, 965 (63.7%) self-identified as women, 546 (36.1%) as men, and 3 (0.2%) as nonbinary. Participants were mostly female at birth (n=967, 63.7%). Most participants were unemployed (n=856, 55.5%) and had a postsecondary education (n=1128, 74.5%). In terms of pain frequency, most (n=924, 59.8%) participants reported intermittent pain, and 378 (28.4%) reported pain duration of 10 years or more.

**Table 1.** Characteristics of the sample (N=1549).

Characteristics	Descriptive statistics
Age (y), mean (SD)	61.1 (14.4)
Age categories (y), n (%)	
18‐34	80 (5.2)
35‐54	379 (24.5)
≥55	1090 (70.4)
Self-identified gender, n (%)	
Woman	965 (63.7)
Man	546 (36.1)
Other	3 (0.2)
Sex at birth, n (%)	
Female	967 (63.7)
Male	551 (36.3)
Employed, n (%)	
Yes	687 (44.5)
No	856 (55.5)
Postsecondary education, n (%)	
Yes	1128 (74.5)
No	386 (25.5)
Pain pattern, n (%)	
Constant	622 (40.2)
Occasional	924 (59.8)
Pain duration categories, n (%)	
3‐11 months	241 (18.1)
1‐4 years	505 (38.0)
5‐9 years	206 (15.5)
≥10 years	378 (28.4)
PROMIS^a^ Pain Interference score, mean (SD)	8.6 (4.1)
Number of comorbidities, mean (SD)	1.9 (1.6)
Multimorbidity (coexistence of 2 or more chronic illnesses), n (%)	
Yes	724 (52.8)
No	648 (47.2)

a PROMIS: Patient-Reported Outcomes Measurement Information System.

Table 2 presents social media use variables, in general and across sex groups. Most participants reported using social media (n=1308, 86.4%; 95% CI 84.7‐88.1; 35 missing). Such numbers were higher among females (n=853, 88.9%) than among males (n=446, 81.7%). For reference, numbers for the whole CEMPUS cohort, including people without chronic pain, are also presented in the table; these numbers were very similar. Among our sample of people living with chronic pain, the average time spent on social media per day to directly connect with people was 39.3 (SD 62.9) minutes. As for the use of social media for recreational purposes without directly interacting with people, the average time spent was 73.8 (SD 84.1) minutes, and for professional purposes, it was 15.3 (SD 43.9) minutes. The mean BSMAS score among participants was 8.5 (SD 3.3), and 27 (1.8%) participants had a score of 19 or above (indicating problematic social media use).

**Table 2.** Social media variables for people living with chronic pain.

Social media variables	In general	Among females	Among males
Social media use among our sample of people living with chronic pain, n (%)	1308 (86.4)	853 (88.9)	446 (81.7)
Social media use in the whole CEMPUS^a^ cohort (for reference), n (%)	2437 (86.6)	1519 (88.8)	904 (83.0)
Average time spent per day on social media to directly connect with people (min)			
Mean (SD)	39.3 (62.9)	41.2 (53.7)	35.5 (76.4)
Median (IQR)	20.0 (0-60)	30.0 (5-60)	10.0 (0-60)
Average time spent per day on social media for recreational purposes without directly interacting with people (min)			
Mean (SD)	73.8 (84.1)	76.6 (84.5)	68.0 (82.9)
Median (IQR)	60.0 (10-120)	60.0 (15-120)	60.0 (0-120)
Average time spent per day on social media for professional purposes (min)			
Mean (SD)	15.3 (43.9)	15.2 (41.6)	15.6 (48.0)
Median (IQR)	0.0 (0-5)	0.0 (0-2)	0.0 (0-5)
BSMAS^b^ score			
Mean (SD)	8.5 (3.3)	8.7 (3.3)	8.1 (3.2)
Median (IQR)	7.0 (6-10)	7.0 (6-11)	6.0 (6-9)
Problematic social media use (BSMAS score ≥19/30), n (%)	27 (1.8)	16 (1.7)	10 (1.8)
Problematic social media use (BSMAS score ≥24/30), n (%)	1 (0.1)	1 (0.1)	0 (0.0)

a CEMPUS: Cohort as Part of Undergraduate Medical Studies at the University of Sherbrooke.

b BSMAS: Bergen Social Media Addiction Scale.

Table 3 presents the prevalence of social media use across age groups. Participants aged 18 to 34 years and those aged 35 to 54 years showed a similar prevalence of social media use (74/76, 97.4% for 18-34 years; 358/370, 96.8% for 35-54 years of age). The age group of 55 to 64 years showed a slightly lower prevalence of social media use (306/335, 91.3%), and a further drop in prevalence was observed in the age group of 65 to 74 years (386/463, 83.4%). In people aged 75 years or older, the number dipped to 68.1% (184/270). Finally, the prevalence of social media use among participants reporting pain for 10 years or more was 81.3% (300/369) and 88.7% (828/934) among those reporting pain for less than 10 years.

**Table 3.** Social media use among people living with chronic pain according to age categories.

Age categories (y)	Social media use, n/N (%)
18‐34	74/76 (97.4)
35‐54	358/370 (96.8)
55‐64	306/335 (91.3)
65‐74	386/463 (83.4)
75 and older	184/270 (68.1)

In terms of prevalence of different social media platforms used at least once a week in the past year, Facebook (n=1134, 76.5%) and Messenger (n=944, 63.7%) were the most frequently used platforms, followed by YouTube (n=506, 34.1%). The complete portrait of specific social media platforms used is provided in Multimedia Appendix 1. Regarding the 3 most popular platforms, the results were the same among females and males. Across age categories, Facebook, Messenger, and YouTube were also the most used platforms in people aged 35 to 54 years and 55 years and over. However, Instagram was more popular than YouTube among people aged 18 to 34 years (47/74, 63.5% vs 38/74, 51.4%), but it was not more frequently used than Facebook or Messenger.

Table 4 presents the multivariable logistic regression model performed to investigate the associations between different factors and social media use among people living with chronic pain. Independent of other variables included in the model, the factors associated with higher odds of using social media were (1) being a female (aOR 1.96, 95% CI 1.39‐2.77), (2) having a postsecondary education (aOR 1.57, 95% CI 1.11‐2.23), (3) reporting occasional alcohol drinking (occasional vs never: aOR 1.64, 95% CI 1.04‐2.57), and (4) reporting difficulties accessing health care or advice (aOR 1.50, 95% CI 1.01‐2.22). The factors associated with decreased odds of using social media were (1) being older (aOR 0.94, 95% CI 0.92‐0.96), (2) reporting having chronic pain for 10 years or more (aOR ≥10 vs <1 y=0.55, 95% CI 0.31‐0.96), and (3) reporting higher depression levels based on the HADS score for depression (aOR 0.92, 95% CI 0.87‐0.99).

**Table 4.** Results of the bivariable and multivariable analysis intended to identify factors associated with social media use among people living with chronic pain.

Characteristics	Crude OR^a^ (95% CI)	Adjusted *P* value	Adjusted OR (95% CI)
Sociodemographic profile			
Age (y)	0.93 (0.91-0.94)^b^	<.001^b^	0.94 (0.92-0.96)^b^
Sex assigned at birth (female vs male)	1.82 (1.35-2.46)^b^	<.001^b^	1.96 (1.39-2.77)^b^
Country of origin (Canada vs other)	1.39 (0.71-2.71)	.34	1.52 (0.64-3.58)
Postsecondary education (yes vs no)	2.27 (1.67-3.08)^b^	.01^b^	1.57 (1.11-2.23)^b^
Employed (yes vs no)	3.99 (2.77-5.74)^b^	.08	1.29 (0.95-2.36)
Household income (higher than the median household income in Quebec vs lower or close)	4.73 (3.88-5.76)^b^	.65	1.10 (0.74-1.62)
Chronic pain characteristics			
Pain frequency (continuous vs intermittent)	0.89 (0.66-1.20)	.19	0.95 (0.66-1.36)
Pain duration (vs 3 to 11 mo)			
1 to 4 years	0.67 (0.39-1.17)	.71	0.89 (0.50-1.61)
5 to 9 years	0.37 (0.21-0.65)^b^	.14	0.62 (0.32-1.17)
10 years and more	0.35 (0.19-0.62)^b^	.04^b^	0.55 (0.31-0.96)^b^
PROMIS^c^ Pain Interference score	1.01 (0.97-1.04)	.27	1.03 (0.98-1.08)
Health profile and lifestyle			
Number of comorbidities	0.81 (0.75-0.88)^b^	.72	0.98 (0.88-1.10)
Anxiety levels (HADS^d^–anxiety score)	1.08 (1.03-1.13)^b^	.87	1.01 (0.95-1.07)
Depression levels (HADS–depression score)	0.98 (0.94-1.03)	.02^b^	0.92 (0.87-0.99)^b^
Current tobacco smoking (yes vs no)	6.32 (5.42-7.37)^b^	.78	0.92 (0.52-1.62)
Alcohol consumption (vs never)			
Occasionally (3 times per month or less)	2.22 (1.47-3.36)^b^	.03^b^	1.64 (1.04-2.57)^b^
Regularly (once a week or more)	1.48 (0.79-2.76)	.79	1.10 (0.55-2.19)
Cannabis smoking in the last year (yes vs no)	2.97 (1.37-6.46)^b^	.34	1.52 (0.64-3.58)
Average time per day spent sitting down (min)	1.04 (0.99-1.09)	.28	1.03 (0.98-1.08)
Sleep difficulties (Insomnia Severity Index)	1.03 (1.00-1.06)	.66	1.01 (0.98-1.04)
Difficulties accessing health care or advice in the past year (yes vs no)	1.77 (1.24-2.52)^b^	.04^b^	1.50 (1.01-2.22)^b^

a OR: odds ratio.

b These values represent statistically significant associations.

c PROMIS: Patient-Oriented Outcomes Measurement Information System.

d HADS: Hospital Anxiety and Depression Scale.

## Discussion

### Principal Findings

The objective of this study was to assess the general use of social media among individuals living with chronic pain and to identify associated factors. To the best of our knowledge, no previous research has specifically examined this topic in this population. The vast majority of participants living with chronic pain (nearly 9 out of 10) reported using social media. This suggests that these platforms may represent valuable tools for recruitment and knowledge mobilization. However, our findings indicate that relying solely on these methods may overlook certain profiles (eg, men, individuals who have not pursued postsecondary education), thus underscoring the importance of diversifying approaches to reach a broader range of individuals and developing strategies tailored to underrepresented groups.

### Prevalence of Social Media Use

This study found a prevalence of general social media use of 86.4% (1308/1514) among people living with chronic pain. Prevalence estimates of general social media use in people living with chronic pain had not been previously documented. However, the frequency of social media use for specific aims (ie, obtaining health information, self-management of pain, seeking a diagnosis of endometriosis, or learning about the condition, or using digital health resources) was reported to range from 38.1% to 90% in populations with chronic pain [20-23]. The prevalence of general social media use in the general population varies from 58.7% to 100% [60-66]. Variability in prevalence data is likely attributable to the characteristics of the study populations (eg, age and socioeconomic status), the country where the study took place (eg, internet accessibility, cultural norms, and digital literacy), the study recruitment methods, and the social media platforms targeted by the study.

### Factors Associated with Social Media Use

This study investigated the factors associated with general social media use among people living with chronic pain. To date, no study has investigated these factors in a chronic pain population. The results of the multivariable logistic regression suggest that sex and employment were associated with higher odds of reporting social media use. In the general population and populations in pain, studies suggest that females use social media more than males [22,23,60,61,63,64]. This finding is consistent with previous studies reporting differences between women and men in various behaviors and psychosocial characteristics [67]. Furthermore, in the workplace, women tend to spend more time in front of computers than men [68]. However, the present study was not designed to investigate the mechanisms underlying this association.

Our finding regarding the prevalence of general social media use across age groups (68.1% in adults aged ≥75 y, 83.4% in those aged 65‐74 y, and more than 90% in adults aged ≤64 y) suggests that older adults can be reached through social media, but to a lesser extent than younger people. Although the per-year effect size appeared modest (aOR 0.94, 95% CI 0.92-0.96), the cumulative impact over a 10-year age difference corresponds to approximately 46% lower odds of social media use. In our study, the prevalence of general social media use in people living with chronic pain was similar to the age-stratified prevalence reported for Quebec internet users (eg, 55-64 y, 93%; 65-74 y, 92% [60]). The lesser use of social media among individuals in later life aligned with our expectations based on what is known in the general population [60]. Among people living with chronic pain, age has also been found to be associated with a lower probability of social media use to obtain health information [22,23]. This finding is possibly due to intergenerational differences. In fact, unlike younger generations, older generations did not grow up with such technology. This might be associated with the level of comfort of older adults regarding the use of social media. Older adults in the general population have also been found to report less trust in news from social media than younger people [60], thus underscoring another difference between generations. From a recruitment perspective, these findings suggest that complementary outreach approaches may be particularly important when seeking to engage older adults living with chronic pain.

Independent of sex, age, income, or employment, individuals without postsecondary education used social media less. This potential bidirectional association may reflect several factors, including digital literacy, access to technology, differing information-seeking habits, or exposure to online social networks during educational and professional experiences. From a recruitment perspective, this finding suggests that social media–based strategies may be less effective in reaching individuals without postsecondary education. Combining social media with additional outreach channels may therefore help improve inclusiveness and reduce the risk of underrepresenting this subgroup.

Participants were asked to report how often they consumed 5 or more alcoholic beverages on a single occasion. Their consumption was classified as never, occasionally (3 times per month or less), or regularly (once a week or more). Participants who occasionally consumed alcohol were more likely to report social media use than those who never consumed alcohol. The reasons underlying this association remain unclear, particularly given that no similar association was observed among regular alcohol consumers. This finding should therefore be interpreted cautiously. Previous studies conducted in adolescent and adult populations have reported associations between social media use and alcohol consumption [69,70], although the mechanisms underlying these relationships remain debated. As the present study was cross-sectional, the temporal ordering and directionality of the observed association cannot be determined. Additional research is needed to better understand the nature of the relationship between alcohol consumption patterns and social media use among people living with chronic pain.

Our study suggests that individuals reporting difficulties in accessing health care or advice had higher odds of reporting social media use. The direction of this relationship remains uncertain, as individuals with unmet health care needs may be more likely to seek information, advice, or support through social media [71], whereas greater engagement with social media may also influence how health care needs are perceived and addressed.

Independent of age and other variables in the multivariable model, participants reporting a pain duration of 10 years or more were less likely to report social media use than those reporting a pain duration of 3 to 11 months. One possible interpretation is that individuals with longer pain duration may differ from those with more recent pain in their patterns of social media use, information seeking, or support seeking. These results are consistent with prior research, indicating that people living with pain for more than 10 years also report better pain relief [72]. However, the direction of these relationships cannot be established in the present study.

The last statistically significant association identified in our study was between higher depression levels and lower odds of reporting social media use. This finding contrasts with studies conducted in the general population, where social media use has been associated with increased odds of depression [73] and problematic social media use has been positively associated with depression and anxiety [74]. The reasons underlying this discrepancy remain unclear. Previous studies have proposed several mechanisms linking depression and social media use, including differences in motivation, energy levels, concentration, and responses to social comparison processes [75,76]. However, the cross-sectional design of the present study does not permit the determination of the temporal ordering of the observed relationship. Consequently, the association may reflect differences in social media use among individuals with varying levels of depressive symptoms, effects of social media use on depressive symptoms, or more complex bidirectional relationships.

### Recommendations

Although causal relationships cannot be inferred from our findings, the identified associations nevertheless provide practical insights into which subgroups may be more or less likely to be reached through social media–based recruitment and knowledge mobilization strategies. First, researchers and clinicians should recognize that social media are widely used among people living with chronic pain, making them a potentially valuable avenue for communication, engagement, and dissemination of health information. However, since not every person living with chronic pain is a social media user, this method should be part of a broader outreach strategy. When using social media, it would be important to prioritize platforms with higher usage prevalence across all age and sex groups. Based on our results, Facebook and Instagram are relevant options. As social media platforms vary in the demographics they engage, the target group should always be considered when deciding which social media platform to use for recruitment or knowledge mobilization. The development of targeted outreach strategies for groups less likely to report social media use (including older adults, men, individuals without postsecondary education, those reporting higher depression levels, and those living with chronic pain for many years) is important to ensure inclusive recruitment and knowledge mobilization. Further research is needed to evaluate strategies for engaging underrepresented groups identified in this study and to better understand the barriers to social media use within these subgroups. Future research should also directly compare the effectiveness and costs of different recruitment and dissemination channels across population subgroups.

### Strengths and Limitations

This study has several strengths that should be recognized. First, the study design permitted the recruitment of a substantial sample of people living with chronic pain (n=1549) without geographic constraints in the province. The participants in the CEMPUS sample were comparable to probabilistic samples of Canadians living with chronic pain in terms of sex, employment, and postsecondary education [33-35]. Second, our study used a widely accepted definition of chronic pain in addition to validated and recognized scores to measure variables such as pain interference, severity of insomnia, depression and anxiety levels, and problematic social media use.

Some limitations should be noted. An important consideration when interpreting the present findings is that social media can be operationalized in different ways depending on the conceptual framework adopted. While the broad definition used in this study encompasses platforms used for communication, content sharing and consumption, and social or professional networking, some platforms (particularly messaging applications such as Messenger) are not included in all definitions of social media. Furthermore, these platforms may differ substantially in their primary functions, patterns of use, and potential implications for health and well-being. Consequently, the associations observed in this study should not be assumed to apply equally across all social media platforms or forms of engagement. Nevertheless, the inclusion of Messenger in our operational definition of social media is unlikely to have materially affected the results of our primary analysis. The primary outcome was defined as the use of at least one platform, and only 3.8% (57/1482) of participants reported using Messenger without using any other listed platform. Therefore, alternative classifications excluding messaging applications such as Messenger would be expected to have a negligible impact on the observed associations. Future studies should investigate how social media use varies across platforms and according to the purpose and nature of engagement. Examining platform-specific use, frequency of use, health-related vs non–health-related activities, and passive vs active engagement may provide more actionable insights for recruitment strategies and knowledge mobilization initiatives targeting adults living with chronic pain.

The age distribution of the CEMPUS cohort should be considered when interpreting the present findings. Compared with representative samples of adults living with chronic pain, CEMPUS participants tend to be older on average. Because social media use generally decreases with age, the overall prevalence estimate reported in this study may be somewhat conservative. However, in our study, prevalence estimates were also presented separately across age groups, which limit the potential influence of the cohort’s age distribution on these results and allow for more meaningful comparisons across age categories. The implications for the multivariable analyses are likely more limited. By simultaneously considering age alongside other participant characteristics, multivariable models help distinguish factors that are independently associated with social media use from those that merely reflect age-related differences. Indeed, older age was independently associated with lower odds of social media use in the present study. Therefore, although caution is warranted when generalizing the overall prevalence estimate, the identified correlates of social media use are less likely to be explained by the older age profile of the cohort.

Another limitation that precluded the use of problematic social media use as the dependent variable in multivariable analyses was the small number of participants meeting the established cutoff for problematic social media use. Although several associations reached statistical significance in our study, effect sizes and their magnitudes varied. Associations involving depression scores appeared modest on a per-point basis, whereas stronger associations were observed for sex, education level, and chronic pain duration. It should also be noted that pain intensity and digital literacy were not available in the cohort and therefore could not be considered in the analyses. Moreover, CIs for some associations were close to 1, pointing to the necessity of a nuanced interpretation of the findings. In addition, the self-reported nature of the data used in this study raises the possibility of memory and underreporting bias (eg, reported time spent on social media shaped by what participants consider socially acceptable). Finally, the cross-sectional design does not permit causal inference and limits the ability to establish the temporal ordering of several observed associations. For some characteristics, such as age, sex, and educational attainment, reverse causation is unlikely. However, for other factors identified in this study, including depression levels, alcohol consumption, and difficulties accessing health care or advice, the direction of the observed relationships remains uncertain and may be bidirectional. Consequently, the findings should be interpreted as hypothesis-generating rather than causal. Future longitudinal studies are needed to better understand how social media use and health-related characteristics evolve over time and to clarify the directionality of these associations.

### Conclusion

General social media use was highly prevalent in our sample of adults living with chronic pain, supporting the potential of these platforms for recruitment, participant engagement, and knowledge mobilization initiatives. Facebook, Messenger, and YouTube represent the most used platforms among this population. However, social media use was not uniform across all subgroups. Older adults, individuals living with chronic pain for a longer duration, and those reporting higher levels of depression were less likely to report social media use. These findings suggest that while social media may represent a valuable component of outreach strategies targeting people living with chronic pain, relying on social media alone may contribute to the underrepresentation of certain groups. Combining social media with complementary recruitment and dissemination approaches may therefore help promote more inclusive engagement.

## Supplementary material

10.2196/88412 Multimedia Appendix 1 Social media platforms used at least once a week in the last 12 months among people living with chronic pain.

## Abbreviations

aOR: adjusted odds ratio
BSMAS: Bergen Social Media Addiction Scale
CEMPUS: Cohort as Part of Undergraduate Medical Studies at the University of Sherbrooke
CHERRIES: Checklist for Reporting Results of Internet E-Survey
HADS: Hospital Anxiety and Depression Scale
PROMIS: Patient-Reported Outcomes Measurement Information System
STROBE: Strengthening the Reporting of Observational Studies in Epidemiology
WHO: World Health Organization
