# Supplementary material for: Social Media Use Among People Living with Chronic Pain: Cross-Sectional Study of Associated Factors
Source: J Med Internet Res. 2026 Sep 4;28:e88412. doi: 10.2196/88412 (PMC13544617; doi:10.2196/88412)
Supplement: Supplementary file 1 [file jmir-v28-e88412-s001.pdf]

Multimedia Appendix 1

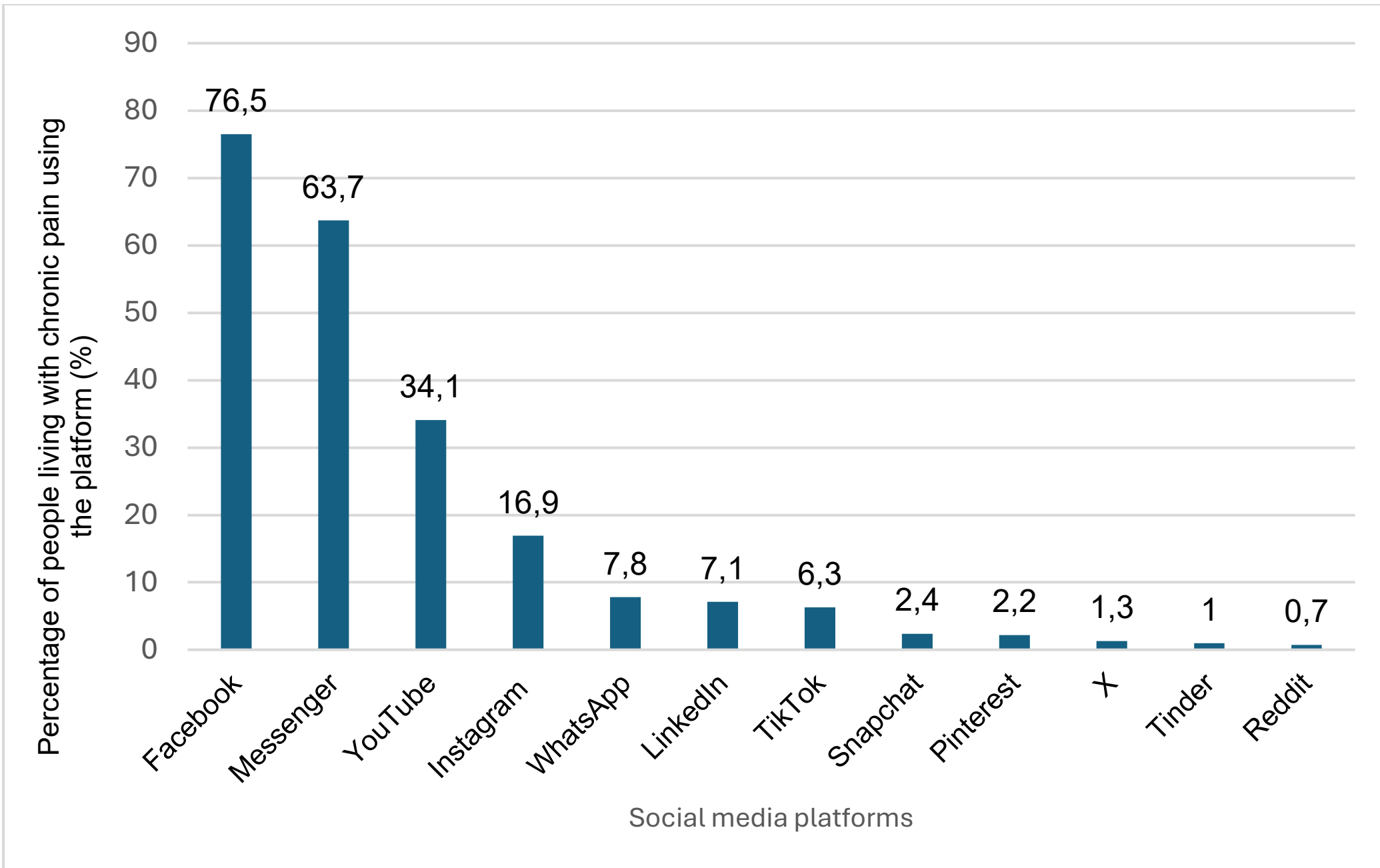

Social media platforms used at least once a week in the last twelve months among people living with chronic pain.
